# Supplementary figures and images for: CXCR3 Inhibition Blocks the NF-κB Signaling Pathway by Elevating Autophagy to Ameliorate Lipopolysaccharide-Induced Intestinal Dysfunction in Mice
Source: Cells. 2023 Jan 1;12(1):182. doi: 10.3390/cells12010182 (PMC9818741; doi:10.3390/cells12010182)

**A**

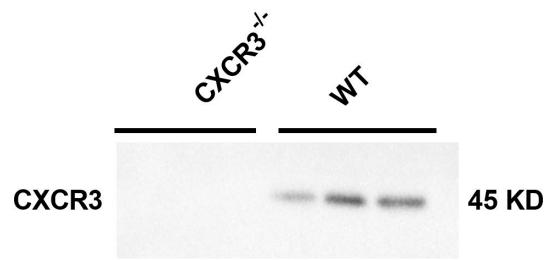

**B**

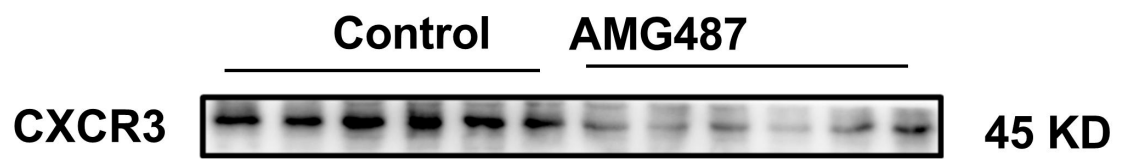

**Figure S1.** CXCR3 expression in CXCR3<sup>-/-</sup> mice (A) and IPEC-J2 cells (B).

Supplement: Supplementary file 1 [file cells-12-00182-s001.zip › cells-2067494 supplementary.pdf]
